# Pressure RElieving Support SUrfaces: a Randomised Evaluation 2 (PRESSURE 2): study protocol for a randomised controlled trial

**DOI:** 10.1186/s13063-016-1703-8

**Published:** 2016-12-20

**Authors:** Sarah Brown, Isabelle L. Smith, Julia M. Brown, Claire Hulme, Elizabeth McGinnis, Nikki Stubbs, E. Andrea Nelson, Delia Muir, Claudia Rutherford, Kay Walker, Valerie Henderson, Lyn Wilson, Rachael Gilberts, Howard Collier, Catherine Fernandez, Suzanne Hartley, Moninder Bhogal, Susanne Coleman, Jane E. Nixon

**Affiliations:** 1 Clinical Trials Research Unit, Leeds Institute of Clinical Trials Research, University of Leeds, Leeds, LS2 9JT UK; 2 Academic Unit of Health Economics, Leeds Institute of Health Sciences, University of Leeds, Leeds, LS2 9JT UK; 3 Leeds Teaching Hospitals NHS Trust, Leeds, LS9 7TF UK; 4 Leeds Community Healthcare NHS Trust St Mary’s Hospital, Leeds, LS12 3QE UK; 5 School of Healthcare, University of Leeds, Leeds, LS2 9JT UK; 6 Quality of Life Office, Psycho-oncology Co-operative Research Group, School of Psychology, University of Sydney, Level 6 North, Chris O’Brien Lifehouse (C39Z), Sydney, NSW Australia; 7 Mid Yorkshire Hospitals NHS Trust, Dewsbury, WF13 2 UK; 8 Pressure Ulcer Research Service User Network, Leeds, LS2 9JT UK; 9 Northumbria Healthcare NHS Foundation Trust, North Shields, NE29 8NH UK

**Keywords:** Pressure ulcers, Double triangular group sequential design, Randomised controlled trial, Alternating pressure mattress, Standard foam mattress, Quality of life

## Abstract

**Background:**

Pressure ulcers represent a major burden to patients, carers and the healthcare system, affecting approximately 1 in 17 hospital and 1 in 20 community patients. They impact greatly on an individual’s functional status and health-related quality of life. The mainstay of pressure ulcer prevention practice is the provision of pressure redistribution support surfaces and patient repositioning. The aim of the PRESSURE 2 study is to compare the two main mattress types utilised within the NHS: high-specification foam and alternating pressure mattresses, in the prevention of pressure ulcers.

**Methods/Design:**

PRESSURE 2 is a multicentre, open-label, randomised, double triangular, group sequential, parallel group trial. A maximum of 2954 ‘high-risk’ patients with evidence of acute illness will be randomised on a 1:1 basis to receive either a high-specification foam mattress or alternating-pressure mattress in conjunction with an electric profiling bed frame. The primary objective of the trial is to compare mattresses in terms of the time to developing a new Category 2 or above pressure ulcer by 30 days post end of treatment phase. Secondary endpoints include time to developing new Category 1 and 3 or above pressure ulcers, time to healing of pre-existing Category 2 pressure ulcers, health-related quality of life, cost-effectiveness, incidence of mattress change and safety. Validation objectives are to determine the responsiveness of the Pressure Ulcer Quality of Life-Prevention instrument and the feasibility of having a blinded endpoint assessment using photography. The trial will have a maximum of three planned analyses with unequally spaced reviews at event-driven coherent cut-points. The futility boundaries are constructed as non-binding to allow a decision for stopping early to be overruled by the Data Monitoring and Ethics Committee.

**Discussion:**

The double triangular, group sequential design of the PRESSURE 2 trial will provide an efficient design through the possibility of early stopping for demonstrating either superiority, inferiority of mattresses or futility of the trial. The trial optimises the potential for producing robust clinical evidence on the effectiveness of two commonly used mattresses in clinical practice earlier than in a conventional design.

**Trial registration:**

ISRCTN01151335. Registered on 14 May 2013. Protocol version: 5.0, dated 25 September 2015

Trial sponsor: Clare Skinner, Faculty Head of Research Support, University of Leeds, Leeds, LS2 9JT; 0113 343 4897; C.E.Skinner@leeds.ac.uk.

## Background

Pressure ulcers (PUs) represent a major burden to patients, carers and the healthcare system affecting approximately 1 in 17 hospital and 1 in 20 community patients [1–3]. They impact greatly on an individual’s functional status and health-related quality of life as a consequence of distressing symptoms including pain, exudate and odour, increased care burden, prolonged rehabilitation, requirement for bed rest, hospitalisation and for those who work, prolonged sickness absence [4, 5].

The primary cause of a PU is mechanical load in the form of pressure or pressure and shear, applied to soft tissues, generally over a bony prominence. They range in severity from non-blanchable erythema (Category 1), superficial skin loss (Category 2) to severe ulcers involving fat, muscle and bone (Category 3, 4 or unstageable) [6].

PU development is influenced by the intensity and duration of pressure and occurs when the soft tissues are unable to tolerate the sustained mechanical loads that develop between bony prominences and a support surface e.g. the sacrum and a mattress [7]. They are a cross-specialty problem, a complication of serious acute or chronic illness in patient populations characterised by high levels of co-morbidity and mortality [8, 9].

The mainstay of PU prevention practice is the provision of pressure redistribution support surfaces (mattresses, cushions) and patient repositioning, to minimise both the intensity and duration of pressure exposure of vulnerable skin sites, not adapted to sustained and/or excessive loading [6, 7]. Pressure-relieving mattresses either distribute the patient’s weight over a larger contact area providing ‘constant low pressure’ or they mechanically vary the pressure beneath the patient, so reducing the duration of the applied pressure [10].

The two main mattress types utilised within the National Health Service (NHS) are *high-specification foam* (HSF) mattresses, which are classified as a ‘low-tech constant low-pressure device’ and *alternating-pressure mattresses* (APMs), which are classified as ‘high-tech’ support surfaces [10, 11]. Alternating pressure mattresses are electrically powered and comprise large air-filled pockets which inflate and deflate in cycles.

The conclusions of the updated Cochrane systematic review [11] did not differ from the previous review [10]. The recent review concluded that foam alternatives to standard hospital foam mattresses reduce the incidence of pressure ulcers in patients at risk (RR 0.40 95% CI 0.21 to 0.74) [11]. One trial suggested that alternating-pressure mattresses may be more cost-effective than alternating-pressure overlays in a UK context [11]. However, the relative merits of APMs and constant low-pressure devices remain unclear and recommended the evaluation of APMs compared to constant low-pressure devices such as HSF [11].

There is evidence that some patients do not like APMs [12–14]. The alternating sensation is disliked by some patients and can cause feelings of nausea and impact upon sleep. In addition, upon patient movement, the air-filled pockets are compressed and patients find it difficult to mobilise in bed and also report feeling unstable at the mattress edge, either when they are getting in and out of bed or feeling like they will be ‘rolled out of bed’, creating an unsafe feeling [4, 12–14]. Other issues include noise from the pump, technical failure and attendant alarms.

The National Institute for Health and Care Excellence (NICE) guidance recommends the use of an HSF mattress in adults who are admitted to secondary care and who are assessed as being at high risk of developing a PU in primary and community care settings [7]. HSF mattresses are also recommended for adults with a PU, and if this is not sufficient to re-distribute pressure, to consider the use of a dynamic support surface [7]. Therefore the NICE guidance states that HSF is the recommended ‘minimum’ standard of care for mattress provision in the prevention and treatment of PUs. Despite the large difference in the unit cost with HSF units ranging from £180 to £600 and APM units ranging from £1000 to £5000 and limited evidence of benefit of APMs compared to HSF in PU prevention, they are in widespread clinical use. This has been evidenced in the National Institute of Health Research (NIHR) PURPOSE pain cohort study where mattress allocation by ward staff to a high-risk target population (mobility impaired and/or Category 1 PU and/or pressure area-related pain) was 48% HSF: 52% APM [15, 16], reflecting a lack of standardised practice and clinical uncertainty relating to mattress provision for ‘high-risk’ patients.

In light of the priority being given to PU prevention by the NHS [7], the high cost and lack of evidence relating to the effectiveness of mattresses in common use in the NHS, ad hoc practice in mattress allocation and the disadvantages and difficulties reported by patients in the use of APMs, we are undertaking a randomised controlled trial (RCT) to compare HSF and APMs in a high-risk in-patient population.

### Objectives

The main aim of the study is to determine the clinical and cost effectiveness of high-specification foam (HSF) and alternating-pressure mattresses (APM) when both are used in conjunction with an electric profiling bed frame in secondary care and community in-patients facilities in patients with evidence of acute illness, for the prevention of Category 2 (and above) PU.

The primary objective is to compare the time to developing a new Category 2 or above PU, in patients using HSF to those using APM by 30 days post end of treatment phase.

The secondary objectives are:

1. To compare the time to developing a new Category 3 or above PU, between patients using HSF and those using APM
2. To compare the time to developing a new Category 1 or above PU, between patients using HSF and those using APM
3. To compare the time to healing of pre-existing Category 2 PU between patients using HSF and those using APM
4. To determine the impact of HSF and APM on health-related quality of life
5. To determine the incremental cost-effectiveness of APM compared to HSF from the perspective of the health and social care sectors
6. To compare incidence of mattress change between patients using HSF and those using APM
7. To compare safety between patients using HSF and those using APM

### Secondary validation objectives

1. To determine responsiveness of the Pressure Ulcer Quality of Life-Prevention (PU-QoL-P) instrument
2. To assess the feasibility of a blinded outcome assessment using photography and central blinded endpoint review

## Methods/Design

The PRESSURE 2 trial is a multicentre, open, randomised, double triangular group sequential, parallel group trial, with two planned interim analyses and validation substudies.

**Fig. 1:**
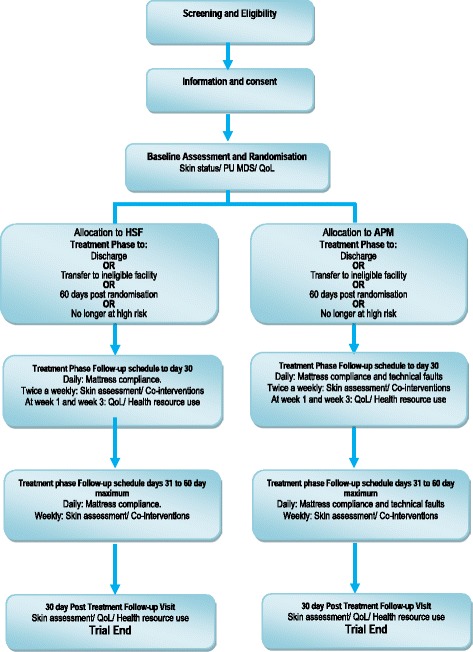
Trial flow diagram

A maximum of 2954 ‘high-risk’ patients with evidence of acute illness will be randomised to receive either HSF or APM in conjunction with an electric profiling bed frame from randomisation to the end of the treatment phase (maximum 60 days), with a final assessment at 30 days post the end of treatment phase (Fig 1).

### Study population

The multicentre trial will be conducted in acute secondary care hospitals, community hospitals and NHS-funded intermediate care/rehabilitation facilities in England and Scotland. All participating centres have Tissue Viability Nurse Specialists in post with hospital-wide remits to establish pressure ulcer prevention guidelines, policies and practice. Patients with evidence of acute illness will be assessed for eligibility in accordance with the criteria in Table 1.

**Table 1:** Inclusion and exclusion criteria

Inclusion criteria
1. Evidence of acute illness through:
a. acute admission to secondary care hospital, community hospital or NHS-funded intermediate care/rehabilitation facility
b. secondary care, community hospital or NHS-funded intermediate care/rehabilitation facility in-patient with onset of acute illness secondary to elective admission
c. recent secondary care hospital discharge to community hospital or NHS-funded intermediate care/rehabilitation facility
2. Aged ≥ 18 years
3. Have an expected total length of stay of 5 or more days
4. At high risk of PU development due to one or more of the following:
a. bedfast/chairfast AND completely immobile/very limited mobility (Braden activity score 1 or 2 and Mobility score 1 or 2) [20]
b. Category 1 PU on any pressure area skin site
c. localised skin pain on a healthy, altered or Category 1 pressure area skin site
5. Consent to participate (written informed consent, witnessed verbal consent, consultee agreement (England) or nearest relative/guardian/welfare attorney (Scotland))
6. Expected to comply with the follow-up schedule
7. The patient is on an electric profiling bed frame
Exclusion criteria
1. Have previously participated in the PRESSURE 2 trial
2. Have a current or previous Category ≥3 PU
3. Have planned admission to ICU where standard care is alternating-pressure mattress provision
4. Unable to receive the intervention (for example, sleep at night in a chair or unable to be transferred to randomised mattress)
5. Patient weight is lower or higher than weight limits for HSF and alternating-pressure mattresses (<45 kg/>180 kg)
6. It is ethically inappropriate to approach the patient

*HSF* high-specification foam, *ICU* intensive care unit, *NHS* National Health Service, *PU* pressure ulcer

## Consent

Where eligibility is indicated by the attending clinical team, patients will be flagged to a member of the hospital Tissue Viability Team (TVT). A full verbal explanation of the study Patient Information Leaflet (PIL) will be provided by a member of the TVT for the patient to consider. Following information provision, patients will have as long as they need to consider participation and will be given the opportunity to discuss the study with their family and other healthcare professionals before they are asked whether they would be willing to participate in the study. Assenting patients will be formally assessed for eligibility and invited to provide informed consent.

For patients capable of giving consent but physically unable to complete the written aspects of the consent form, witnessed consent will be provided by a family member or friend of the patient, or another member of the patient’s healthcare team who is not directly involved in the research study using the Witnessed Consent Form.

A large proportion of patients at risk of pressure ulcers have cognitive impairment affecting their understanding of the trial and/or dementia. Cognition impacts upon compliance with repositioning and self-care in the use of the electric profiling functionality, which is an integral component of the intervention package. It is important that the trial population is representative of the normal NHS patient population and therefore patients who lack capacity will also be recruited onto the trial.

The assessment of capacity will relate specifically to decisions pertaining to this particular research project. Each patient will be assumed to have capacity unless it is established that they lack capacity. Ward-based nurses identifying patients for study participation will be asked to consider aspects of capacity before any approach to patients is made and during the information-giving stage prior to consent. The TVT member will assess the patient’s ability to understand what decisions they need to make and why; the consequences of the decision to participate; their ability to understand, use and retain the information related to the decision to participate and be able to communicate their decisions effectively (as specified in the Mental Capacity Act 2005). If there is any concern about capacity the ward nurse/TVT member will consult further with other members of the attending clinical team and/or relative/carer/friend (as appropriate) and a decision will be made with the relative/carer/friend as to whether the patient is able to provide written consent. Where the patient is thought not to have capacity to consent, a relative, carer or friend who is interested in the patient’s welfare will act as a personal consultee or nearest relative/guardian/welfare attorney (Scotland).

The relative/carer/friend will be involved in the information and decision-making process with the patient and will advise the TVT member on their presumed wishes and feelings and consultee or nearest relative/guardian/welfare attorney (Scotland) assent will be obtained on behalf of the patient. The relative, carer or friend will be advised to set aside their own views and provide advice on the participation of the patient in the research, taking into consideration the patient’s wishes and interests. Patients will not be required to do anything which is contrary to any advance decisions or statements that have been made by them in relation to their treatment or any other matter. Advance decisions made by the patient about their preferences and wishes will always take precedence.

If, despite taking all reasonable steps, a personal consultee cannot be identified and contacted then a nominated consultee or nearest relative/guardian/welfare attorney (Scotland) would be approached. This person would have no connection with the research project. They would be nominated by the TVT member; they would most likely be the patient’s lead clinician. The consultee or nearest relative/guardian/welfare attorney (Scotland) would be provided with the information leaflet describing the research study and the role of the consultee/nearest relative/guardian/welfare attorney and it would be emphasised that they are being asked to act on behalf of the patient, rather than on any personal views or feelings.

Where a patient has been enrolled into the study after consultee or nearest relative/guardian/welfare attorney (Scotland) agreement and they subsequently regain capacity, a member of the research team will discuss the study with the patient; provide a full verbal explanation of the trial and a Patient Information Leaflet explaining the trial and their participation in the trial. Assenting patients will be invited to provide informed consent/witnessed verbal consent for ongoing participation. Those who do not wish to continue in the trial will be withdrawn.

Consent for photographs to be taken of skin sites will be obtained at time of consent to the trial with an opt-out clause allowing patients to withdraw consent for photographs at any time.

Patients who change from their allocated mattress will continue follow-up assessments unless they are unwilling to do so, and patients who withdraw from the trial will continue to be managed in line with standard care. Patients with capacity at study entry who subsequently lose capacity will be withdrawn from the trial.

## Randomisation

Following confirmation of informed consent, eligibility and completion of baseline assessments, patients will be randomised by an authorised member of the research team at the site. Patients will be randomised in a 1:1 allocation ratio, to receive either HSF or APM. Randomisation will be performed using minimisation, incorporating a random element, via a central 24-hour automated telephone randomisation system based at the Leeds Institute of Clinical Trials Research (LICTR). The dynamic allocation method should ensure intervention groups are well balanced for the following minimisation/stratification factors:

- Centre
- PU status (no pressure ulcer, Category 1, Category 2)
- Secondary care hospital, community hospital, intermediate care or rehabilitation facility
- Method of consent (written OR witnessed verbal OR consultee or nearest relative/guardian/welfare attorney (Scotland) agreement)

The randomisation system will include an automated internal check using NHS numbers to confirm that the patient has not been recruited to the trial previously.

In addition, at the time of randomisation, 10% of patients will be randomly selected for expert clinical assessment and photography.

## Interventions

Patients will be randomised to either HSF or APM products used by the participating centre.

Specifications for both HSF and APM have been developed and mattresses are approved for inclusion in the trial based on these specifications, as defined in a PRESSURE 2 Mattress Specification Guideline. The guideline for HSF includes foam density (foam fatigue and foam hardness) and mattress cover characteristics (removable, minimum two-way stretch, vapour-permeable, covered zips) as defined in BS 3379 [17] and for APMs includes minimum and maximum values for cell height, cycle time and cycle frequency [17]. All mattresses will comply with the Medical Devices Regulations SI2002/618. The PRESSURE 2 Mattress Specification Guideline also provides details of excluded mattresses including: low air loss mattresses; combination mattresses e.g. static foam and alternating cells, static foam and gel, hybrid alternating and low air loss; and other continuous static low-pressure mattresses including fibregel, fluid and air filled mattresses.

Trial-approved mattresses are identified at each centre on an ongoing basis. After randomisation an eligible mattress will be sourced and allocated by the Clinical Research Nurse (CRN)/Registered Healthcare Professional (RHCP). Mattress allocation is expected within 24 hours of randomisation.

All patients will have an electric profiling bed frame as an adjunct to the trial mattress.

## Assessments/data collection and follow-up

### Baseline assessment

Patient demographic information including NHS number, date of birth, gender, date of admission, type of admission, category of medical condition (orthopaedics and trauma, oncology, critical care and other conditions) and ethnicity will be recorded.

Patient risk profiles and key risk factors will be recorded through clinical assessment and completion of the Braden scale and PURPOSE-T including baseline skin status, localised skin pain on pressure areas, activity and mobility status, sensory perception, diabetes, conditions affecting macro and micro circulatory function, nutrition, skin moisture, friction and shear, PU history, height and weight (self-report or notes where available) and duration and size of ulcer and photograph (if consented) for patients with a pre-existing category 2 PU [9, 15, 16, 18].

PU prevention interventions pre-randomisation will be recorded including mattress type, turning frequency and seating provision.

Patients will be asked to complete quality of life questionnaires, which include: 12-item Short Form (SF-12), Pressure Ulcer Quality of Life-Utility Index (PUQoL-UI) [15] and EuroQol-5D 5 level (EQ-5D-5L); with the latter two by proxy for patients who lack capacity. In addition PUQoL-Prevention (P) data, an adapted version of PUQoL [19], will be collected in a subset of patients.

It is expected that the baseline assessment and randomisation will take place on the same day.

### Treatment phase follow-up assessment

Treatment phase follow-up assessments will be undertaken up to a maximum of 60 days post randomisation. These will be undertaken by a trained CRN/RHCP twice weekly from randomisation up to 30 days, then once weekly up to 60 days. The treatment phase is defined as the period from randomisation to discharge, transfer to an ineligible facility, when the patient is considered no longer at high risk (considered at each assessment), reaching 60 days, withdrawal or death, whichever is soonest. No longer at high risk is defined as no Category 1 or above PU on any skin site AND no localised skin pain on a healthy, altered or Category 1 skin site, AND improved mobility and activity (Braden activity score 3 or 4 AND Mobility score 3 or 4) [20].

Patients will have a skin assessment, including pain and photography (if consented) of Category 2 and above ulcers where present and not previously photographed, and PU prevention interventions recorded. Mattress compliance and technical faults will be collected daily. At weeks 1 and 3 post randomisation*,* healthcare resource utilisation, and quality of life questionnaires SF-12 [21], PUQoL-UI and EQ-5D-5L [22] will be completed (PUQoL-UI and EQ-5D-5L by proxy for patients lacking consent); the PU-QoL-P will again be administered to a subset of patients.

Following the end of the treatment phase patients will receive care in line with routine standard practice.

### Post-treatment phase follow-up assessment

At 30 days post end of the treatment phase, a final skin assessment (including photography if selected) will be undertaken at the patient’s home or care facility by a member of the research team. This assessment will only be at 90 days if the end of the treatment phase has a duration of 60 days. Should the patient be discharged, considered as no longer at high risk, or transferred to an ineligible facility prior to 90 days then the final follow-up assessment will be scheduled to take place at 30 days post the date of: discharge, no longer at risk or transfer.

Healthcare resource utilisation, and quality of life questionnaires (SF-12, Pressure Ulcer Quality of Life-Utility Index (PUQOL-UI) and EQ-5D-5L; and PU-QoL-P in a subset of patients) will be completed.

**Fig. 2:**
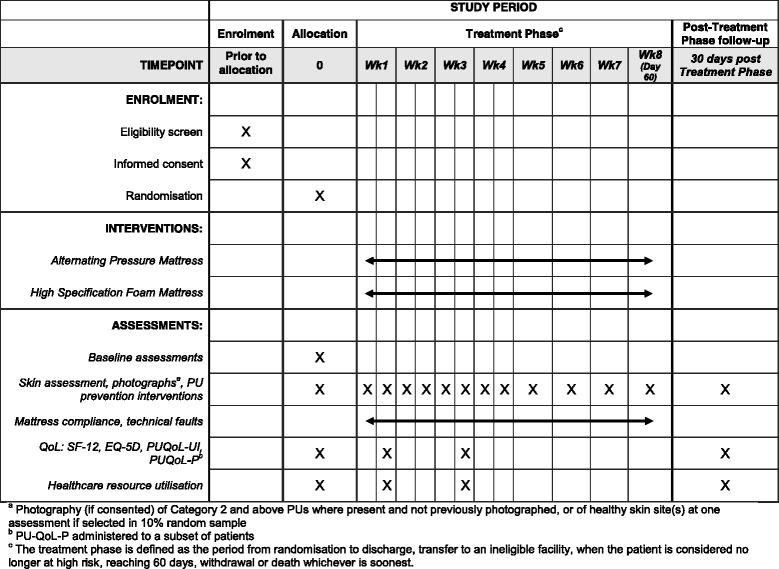
Schedule of enrolment, interventions, and assessments

See Fig. 2, for the schedule of enrolment, interventions, and assessments.

## Endpoints

### Primary endpoint

The primary endpoint is time to developing a new Category 2 or above PU from randomisation to 30 days from the end of the treatment phase (maximum of 90 days).

### Secondary endpoints

The secondary endpoints will be determination of the following:

- Time to developing a PU of Category 3 or above from randomisation to trial completion
- Time to developing a PU of Category 1 or above from randomisation to trial completion
- Time to healing of pre-existing Category 2 PUs from randomisation to trial completion
- Health-related quality of life using SF-12 and PU-QoL-P instruments
- Incremental cost-effectiveness of APM compared to HSF from the perspective of the health and social care sectors using EQ-5D-5L and health and social care resource utilisation questionnaire
- Mattress changes during the treatment phase
- Adverse events

### Skin assessments

Skin will be assessed by trained CRNs/RHCPs using the international PU classification (NPUAP/EPUAP 2014). At baseline and each visit post randomisation, all skin sites at risk of PU development (sacrum, right and left buttocks, hips, heels and elbows), will be assessed by the CRN/RHCP and confirmed as normal skin (0), incontinence-associated dermatitis (IAD), alteration to intact skin (A) or the presence of a PU. PUs when present will be classified and recorded using the National Pressure Ulcer Advisory Panel/European Pressure Ulcer Advisory Panel (NPUAP/EPUAP) classification as Category 1–4 or unstageable [6]. Reasons will be recorded for skin sites that are unable to be assessed.

### Blinding

As it is not possible to blind patients or the TVT, a validation substudy, using photography with blinded central review and expert clinical assessment of the skin sites by an independent member of the team, will be conducted to assess any bias in the reporting of Category 2 or above PUs. Two approaches are being implemented: To assess the risk of over-reporting of the ulcer, the CRN/RHCP who is not independent will photograph each Category 2 or above PU at first observation. To assess risk of under-reporting, 10% of patients are randomised for expert clinical assessment and photography of two skin sites by an independent member (if the patient has at least one PU then a photograph will be taken at a maximum of one PU skin site and one healthy skin site). A standardised photography process will be implemented to ensure a consistent approach is used across all centres when taking and transferring photographs; the central blinded review of the photographs will be undertaken by expert clinical nurse specialists.

### Sample size

A maximum of 588 events i.e. patients developing a new Category 2 or above PU, corresponding to 2954 patients, are required for the study to have 90% power for detecting a difference of 5% in the incidence of Category 2 or above PUs between APM and HSF, assuming an incidence rate of 18% on APM [13, 23] and 23% on HSF [15, 16, 23, 24] (corresponding to a hazard ratio of 0.759), two-sided significance level of 5%, and accounting for 6% loss to follow-up [13].

The PU incidence for APM of 18% was estimated on the intention-to-treat (ITT) population for the completed PRESSURE trial [13], the PURPOSE Pain Cohort study [15, 16] and the trial reported by Vanderwee [23], and hence the sample size estimate incorporates the effect of any non-compliance. The sample size accounts for multiplicity in the interim analyses using Lan-DeMets α and β spending functions [25].

PU incidence rates cannot be estimated accurately for the HSF and the maximum sample size estimate is based on the detection of the smallest clinically relevant difference of 5% (clinical opinion). If the difference is >5% then the trial will have sufficient power to stop early having demonstrated superiority (or inferiority) of the APM; if the difference is <5% then the trial is likely to stop early for futility.

The approximate sample size for the PUQoL-P substudy will be 500 patients.

A maximum of approximately 1653 photographs are expected for the central blinded review, which will enable kappa to be estimated to within a precision of at least ±0.044 (corresponding to the half width of the 95% CI), assuming 65% of photographs are of Category 2 or above PUs and kappa ≥0.5.

### Statistical analysis

Statistical analysis plans for the interim and final analyses will be finalised and signed off before any data analyses are conducted.

All analyses will be on an ITT basis where patients will be analysed according to treatment group randomised to receive. A per protocol population will also be defined, which will include all eligible randomised patients according to the treatment received but will exclude major protocol violations as agreed by the clinical members of the Trial Management Group (TMG).

### Interim analysis and stopping rules

The group sequential design provides an efficient design through the possibility of early stopping ([26, 27]

As this is a group sequential design, the trial will have a maximum of three planned analyses with unequally spaced reviews at event-driven coherent cut-points:

1. The first analysis conducted after 300 events corresponds to the earliest time point at which the trial can be stopped for demonstrating overwhelming evidence of efficacy or futility, and also corresponds to the minimum number of events required for conducting the economic evaluation. The futility boundaries are constructed as non-binding in order for the Data Monitoring and Ethics Committee (DMEC) to overrule a decision of stopping early for futility in the event that a futility boundary is crossed. In the event of the DMEC recommending that the trial is stopped for futility using the pre-defined stopping criteria, an expected value of sample information analysis will be undertaken to assess the value of additional sample information on the effectiveness parameter, to establish whether continuing the trial would be valuable from the NHS decision-makers’ perspective. If the decision to stop is overruled, the stopping boundaries will be adjusted using the “buy-back alpha” approach [28].
2. The second analysis, conducted after 445 events corresponds to the number of expected events required for trial termination under futility (with 434 corresponding to the number of events required for demonstrating superiority or inferiority of APM to HSF).
3. The final review will be conducted after 588 events have occurred.

**Fig. 3:**
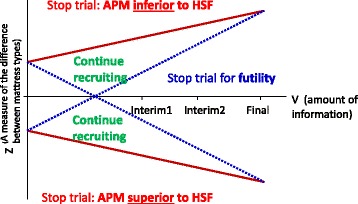
Double triangular group sequential design

The double triangular group sequential design is shown in Fig. 3. The efficacy and futility stopping boundaries are defined using Lan-DeMets α and β spending functions, which provide conservative stop/continue criteria at the interim analyses.

At each interim analysis, the primary analysis of the primary endpoint, time to developing a Category 2 or above PU, will be conducted on both the ITT and per protocol population. The test statistic for the treatment effect from the Cox proportional hazards model (after confirming the proportional hazards assumption is valid), adjusting for the minimisation factors and covariates specified in the primary endpoint analysis section below, will be used to test for a difference between treatment groups. The hazard ratio for the treatment effect and adjusted confidence interval corresponding to the nominal *p* value will be presented.

Interim analyses will be presented to the DMEC who will then advise the Trial Steering Committee (TSC) if there is proof beyond reasonable doubt that the trial should be stopped in accordance with the planned stopping rules.

### Primary endpoint analysis

#### Primary analysis

A Cox proportional hazards model (after confirming the proportional hazards assumption is valid) will be fitted to the primary endpoint, with adjustment for the minimisation factors: healthcare setting, PU status and consent, and the following covariates: presence of pain on a healthy, altered or Category 1 PU skin site, conditions affecting peripheral circulation and treatment group. Centre will be assessed for inclusion as a random effect. The hazard ratio for the estimate of the treatment effect and adjusted confidence interval corresponding to the nominal *p* value will be presented. Patients who do not develop a Category 2 or above PU during the treatment phase or by 30 days post treatment follow-up will be censored at the date of their last evaluable skin assessment.

Kaplan-Meier estimates of the probability of patients developing a Category 2 or above PU over 90 days in each group and adjusted confidence intervals corresponding to the nominal *p* value will be presented.

#### Moderator and mediator analyses

Potential predictors of time to developing Category 2 or above PU will be explored by assessing potential predictor by treatment group interactions in the model. In addition, the relationship between potential moderator and mediator variables (including length of stay, time on allocated mattress, patient turning, and use of specialist cushions, heel protectors and protective dressings) and treatment effect will be modeled.

#### Secondary endpoint analysis

The secondary endpoints, time to developing a PU of at least Category 1, and time to developing a PU of at least Category 3, will be analysed using Cox proportional hazards modelling (after confirming the proportional hazards assumption is valid) adjusting for the minimisation factors and same covariates as for the primary endpoint analysis and treatment group.

A Cox proportional hazards model (after confirming the proportional hazards assumption is valid) will be fitted to the outcome time to healing of pre-existing Category 2 PUs; covariates fitted in the primary endpoint model will be assessed for inclusion and treatment group.

A substudy analysis will be performed to examine the PU-QoL-P instrument responsiveness; effect sizes [29] and standardised response means (SRM) [30] before and after mattress allocation will be estimated. Independent *t* tests will be conducted to assess the ability of the PU-QoL-P instrument to distinguish between patients whose PUs deteriorate or heal. Subject to the results of the instrument responsiveness analysis, QoL domains and subscales for the SF-12 and PU-QoL-P will be compared between treatment groups using multilevel repeated measures modeling allowing for time, mattress type, mattress type by time interaction, adjusting for baseline QoL, patient and patient by time interaction as random effects.

Adverse events and serious adverse events classified as related to the mattress, resulting from administration of any research procedures, and falls and device-related events during the treatment phase and follow-up will be listed and summarised by treatment group.

#### Assessment of the feasibility of the blinded outcome assessment

To assess the feasibility of the blinded outcome assessment using photography and blinded endpoint review, a sensitivity analysis of the primary endpoint analysis will be conducted to assess the risk of over-reporting of category 2 or above PUs, replacing the primary endpoint for those patients with a Category 2 or above PU with the assessment made in the blinded central endpoint review. To assess under-reporting of the development of Category 2 or above PUs, cross-tabulations, kappa and prevalence- and bias-adjusted kappa (PABAK) statistics will summarise the extent of agreement of reporting of Category 2 PU status at skin sites between the independent member of the team, CRN/RHCP and photography assessments.

#### Missing data

The pattern of missing data over time and by treatment group will be assessed. If assumed to be missing at random (MAR), multiple imputation will be considered. If the data are thought to be missing not at random (MNAR) then appropriate methods such as pattern mixture models will be explored.

### Economic evaluation

#### Within-trial economic evaluation

The expected costs and outcomes will be estimated for each treatment group up to 90 days post randomisation; based upon the observed outcomes and resource utilisation collected during the trial. The outcome measure used in the primary economic evaluation will be the quality-adjusted life year (QALY), using utilities derived from the EQ-5D-5L. A secondary analysis will use PUQoL-UI, a condition-specific utility measure derived from the PU-QoL measure [19]. The resource use data collection will focus on those incurred by the NHS including length of stay in hospital, use of hospital outpatient facilities, contact with community-based healthcare services and utilisation of supported living such as care and nursing homes. Unit costs will be obtained from national databases such as the NHS Reference costs [31] and the Personal Social Services Research Unit (PSSRU) costs of health and social care [32]. Other costs will be estimated in consultation with the finance departments of centres recruiting to the trial. Within-trial QALYs and resource cost for a subsample of trial patients will be estimated. Due to the short time horizon for the within-trial analysis, discounting will not be required. Non-parametric bootstrap will be used to estimate the expected costs and outcomes for each group and the associated incremental cost-effectiveness ratio for APM versus HSF. The results of the bootstrap will be used to construct a cost-effectiveness acceptability curve (CEAC), using a range of values of willingness to pay per incremental QALY.

#### Long-term cost-effectiveness of APM versus HSF in prevention of PUs in high-risk patients

A longer-term cost-effectiveness analysis of APM versus HSF will be undertaken. The model will use the perspective of the NHS and outcomes measured using quality-adjusted life years (QALYs). The model will adapt the structure proposed by Padula et al [33]. Data from the trial will be used for transitions between states, costs and utilities. This will include, but not be limited to, probabilities of developing and first and second PU, discharge from hospital and re-admission to hospital; utility values based on the EQ-5D-5L data and costs including daily costs for each type of mattress and extra costs for treatment of PUs. For model parameters for which data could not be collected within the trial we will follow recommended best practice in identifying and synthesising the best available evidence in the literature [34, 35]. Costs and outcomes will be discounted at 3.5% p.a. in line with the NICE recommendations [34]. A probabilistic sensitivity analysis will be undertaken and the results will be reported as a CEAC. The CEAC represents the probability an intervention is cost-effective for a range of willingness to pay per incremental QALY threshold values. Subgroup analyses will be undertaken where analyses of the clinical outcome data suggest a substantial difference in absolute benefit from in a priori identifiable groups.

In the event of an early stopping signal for futility; to assess the value of continuing with the trial from the NHS decision-making perspective, via an expected value of sample information analysis, to inform the deliberations of the DMEC [36].

### Data management

Data will be monitored for quality and completeness. Missing data will be chased until it is received or confirmed as not available. Data received will be linked-anonymised and entered onto a secure database at LICTR in accordance with the 1998 Data Protection Act. Photographs taken will be transferred immediately by secure email to LICTR and stored on a secure database. Once confirmation of receipt is received by the clinical CRN/RHCP, the photographs will immediately be deleted from the camera.

### Trial governance

The TMG, comprising the Chief Investigator, co-investigators, patient representative and project team will meet monthly and is responsible for the clinical set-up, ongoing management and for the interpretation of results. The DMEC, with independent Chair, will meet at least annually to review trial progress, safety and to monitor the overall incidence rate, and following each interim analysis. The TSC, with independent Chair, will meet 6 monthly and provide overall supervision of the project, including trial progress, adherence to protocol and consideration of new information.

## Discussion

The double triangular group sequential trial provides an efficient design through the possibility of early stopping for demonstrating either futility of the trial or superiority of either mattress. The trial optimises the potential for producing robust clinical evidence on the effectiveness of two commonly used mattresses in clinical practice earlier than in a conventional fixed design. A fixed design with the same parameters would require 554 events. Although conducting a group sequential trial increases the maximum sample size required compared to a conventional fixed design, these stopping boundaries will also allow for an increased chance of stopping early. In addition, the trial utilises an early primary endpoint, time to developing a Category 2 or above PU to a maximum of 90 days, and therefore early stopping can be assessed in a timely manner.

On the other hand, a potential limitation of having an early endpoint is that PUs developing over a longer time horizon will be missed, however as PUs generally develop quickly in this patient population [13] this would affect a minority of patients, and it is was therefore considered that the duration of follow-up is optimum to allow early assessment of superiority of either mattress or futility of the trial.

Group sequential trials with a time to event primary endpoint require close monitoring of the overall event rate in order to plan when an interim analysis will be conducted. A benefit of this close monitoring allows the overall event rate to be monitored and evaluated against the assumptions specified in the original trial design, thereby allowing the trial design to be modified prior to an interim analysis if required.

Stopping boundaries provide statistical guidance to the DMEC on the recommendations for stopping the trial early; however factors including patient safety, cost-effectiveness and other information external to the trial will also need to be considered in order to make a fully informed decision on whether or not to continue [28].

As this is a pragmatic trial, the operational specifications for both HSF and APM have been developed and defined in the PRESSURE 2 Mattress Specification Guideline, rather than product-specific mattresses for all trial patients, as this increases the generalisability of the trial findings to the NHS. There are also research costs advantages as the trial does not require the purchasing of specified products.

Having a blinded assessment of outcome measures is a key consideration in reducing the risk of assessment bias. In this study we will assess the feasibility of having a blinded outcome assessment using photography with a blinded independent central review undertaken by clinical nurse specialists. During photograph review sessions, the central panel will be unaware of whether the photographs are to assess under- or over-reporting of Category 2 or above PUs, thereby further mitigating the risk of assessment bias.

A further benefit of this design is the inclusion of the substudy to assess responsiveness of the PU-QoL-P tool at the first planned interim analysis, and which will provide a sufficient number of patients to allow a validation of this psychometric property of the tool.

Findings from the trial will inform future clinical practice in the management of patients at high risk of developing PUs.

### Trial status

The first patient was randomised on 14 August 2013. As of 13 April 2016, 1501 patients have been randomised.

## Abbreviations

APM: Alternating pressure mattress
CEAC: Cost-effectiveness acceptability curve
CRN: Clinical Research Nurse
DMEC: Data Monitoring and Ethics Committee
EPUAP: European Pressure Ulcer Advisory Panel
EQ-5D-5L: EuroQol-5D 5 level
HSF: High-specification foam
IAD: Incontinence-associated dermatitis
ITT: Intention-to-treat
LICTR: Leeds Institute of Clinical Trials Research
MAR: Missing at random
MDS: Minimum data set
MNAR: Missing not at random
NHS: National Health Service
NICE: National Institute for Health Care Excellence
NIHR: National Institute for Health Research
NPUAP: National Pressure Ulcer Advisory Panel
PABAK: prevalence and bias-adjusted kappa
PSSRU: Personal Social Services Research Unit
PU: Pressure Ulcer
PU-QoL-P: Pressure-Ulcer Quality of Life-Prevention
PU-QoL-UI: Pressure Ulcer Quality of Life-Utility Index
QALY: quality-adjusted life year
RCT: randomised controlled trial
RHCP: Registered Health Care Professional
SF-12: 12-item Short Form
SRM: Standardised response mean
TMG: Trial Management Group
TSC: Trial Steering Committee
TVT: Tissue Viability Team
